# Sorghum, or Chinese Sugar Cane

**Published:** 1858-01

**Authors:** 


*Sorghum, or Chinese Sugar Cane.*—This article, which has lately been imported, finds our northern climate peculiarly favorable to its growth. It is now produced in quantities in different parts of the States, and a sample of its product in the form of molasses, has been sent to the Buffalo market. We have lately had an opportunity of testing its properties as an article of food, but the specimen which was shown to us appeared to have been a little burned in the process of manufacture. The peculiarity most interesting to us in a scientific point of view is, that no one has been able to make it granulate and form sugar. We have, indeed, the statement of a distinguished chemist connected with one of our southern sugar works, that this is impossible; this of course would materially injure the prospect of our being relieved from the necessity of depending upon the South for our sugars. We hope that this matter will be thoroughly investigated by our competent chemists, and the substance discovered which prevents the process of granulation common to saccharine matter. When this is accomplished, it seems probable that the sorghum will become an exceedingly important article of commerce, as the cane yields largely, even by the cheap and imperfect processes which have been employed in the manufacture of molasses.

